# Is gestational diabetes mellitus in lean women a distinct entity warranting a modified management approach?

**DOI:** 10.3389/fcdhc.2024.1338597

**Published:** 2024-05-07

**Authors:** Pradnyashree Wadivkar, Meredith Hawkins

**Affiliations:** Global Diabetes Institute, Diabetes Research Center, Albert Einstein College of Medicine, Bronx, NY, United States

**Keywords:** gestational diabetes, lean women, BMI, insulin secretion, GDM, insulin deficiency

## Abstract

During pregnancy, insulin resistance and impaired insulin secretion may lead to the development of Gestational Diabetes Mellitus (GDM). Although a higher Body Mass Index (BMI) is often cited as a risk factor for the development of GDM, lean pregnant women are also at risk of developing GDM based on evidence from several studies. It is proposed that insulin deficiency (more than insulin resistance) leads to the development of GDM in women with low BMI (BMI <18.5 kg/m^2^). Neonates of these women are more at risk of preterm birth and small-for-gestational-age. Given this unique pathophysiology and phenotype, this entity needs a modified management approach. This article aims to raise awareness of GDM in lean women to encourage more research on this topic and create a modified management approach.

## Introduction

Gestational Diabetes Mellitus (GDM) is a type of diabetes with an onset or initial diagnosis during pregnancy, either due to inadequate insulin production or insulin resistance (1). During pregnancy, the placenta secretes hormones such as human placental lactogen, estrogen, progesterone, and cortisol to reduce insulin sensitivity in pregnant women and increase nutrient availability for the fetus (2). In addition, reduced pancreatic β cell mass, reduced β cell number, β cell dysfunction, or a combination of all three factors (which can be either preexistent or directly related to pregnancy, depending on the individual) may contribute to insufficient insulin secretion in pregnancy (3). Thus, insulin resistance and reduced or impaired insulin secretion in pregnancy lead to the development of GDM. Studies have identified multiple potential risk factors for GDM, with higher Body Mass Index (BMI) commonly identified as a risk factor. The association of maternal obesity with insulin resistance and GDM is well established (4, 5), but it is essential to note that there is also evidence suggesting that GDM can occur in lean pregnant women (BMI<18.5 kg/m^2^). Lean pregnant women may have a distinct pathophysiology responsible for the development of GDM, which warrants a modified management approach focused on managing the weight of the mother and fetus as well as achieving the glycemic targets. This article aims to raise awareness of GDM in lean women as a separate entity needing modified management and encourage further scientific research into this field to create specific guidelines for the same.

## Discussion

Evidence from several studies worldwide suggests that GDM is a significant problem among lean women. In a prospective study of pregnant women attending antenatal clinics in the Jammu region of India, the prevalence of GDM in underweight women (BMI <18.5 kg/m^2^) was 3% (6). Yong et al. conducted a retrospective cohort study in 1951 pregnant women registered in the antenatal clinics of Seremban district, Malaysia, to identify the effect of BMI in the first antenatal visit on the risk of GDM. It is interesting to note that in this study, the adjusted Odds Ratio of GDM among pregnant women with BMI <18.5 kg/m^2^ was 0.78 with a confidence interval between 0.47 and 1.31 – thus, even though the overall Odds ratio was <1, the variability within the confidence interval indicates that some women with lower BMI had a higher risk of GDM. Consistent with this intriguing finding, a few studies showed evidence of comparable or even higher risk of GDM in lean women relative to women with normal or higher BMI. Yachi et al. conducted a study to examine the association of BMI at 20 years of age with subsequent risk of GDM in pregnant Japanese women. They found that women who had been lean (with BMI<18 kg/m^2^) at 20 years of age had a 6.3-fold (2.26-17.59) increased risk of developing GDM relative to women with normal BMI at 20 years of age (7). In another study by Inoue et al., in 30 lean Japanese women, nine women (30%) were diagnosed as having GDM (8). A study conducted in Rwanda found that underweight non-diabetic women had 1-hour and 2-hour blood glucose levels comparable to overweight women and higher than women with normal BMI, consistent with the findings in Japan (9). Furthermore, although these studies reported a lower overall prevalence of GDM in lean women, this could still translate into a significant health problem globally, given the absolute number of women at risk of GDM.

### The pathophysiology of GDM among lean women

GDM in lean women may be more related to insulin deficiency than to insulin resistance. Furukawa and Kobayashi examined the correlation between leanness and impaired insulin secretion in pregnant Japanese women. They divided the GDM women in their study into two subgroups based on HOMA-IR (Homeostasis Model Assessment Insulin Resistance): LIR (Low insulin resistance) and HIR (High insulin resistance). After a 75-gram OGTT test, plasma glucose concentrations at 60 and 120 minutes did not differ significantly in the LIR group. In contrast, in the HIR group, the post-glucose load glucose levels were significantly lower at 120 minutes compared to 60-minute levels. The persistent high glucose levels in the LIR group were suggestive of impaired insulin secretion (10). The study found a positive linear correlation between BMI and HOMA-β (Homeostasis model assessment of β-cell function) in women with GDM (10). Also, the authors reported that the HOMA-β of the LIR group was significantly lower than the HOMA-β of the HIR group (10). Based on these findings, they concluded that impaired insulin secretion was strongly related to the onset of GDM in women from the LIR group (10). Additionally, they reported that as the lean Japanese women progressed to worsening glucose tolerance, their insulin secretion was more abnormal relative to insulin resistance. Thus, there may be two distinct causes of GDM in pregnant women at the two extremes of BMI: insulin resistance in overweight and obese women and impaired insulin secretion in lean women (10). Also, Inoue et al. suggested a similar mechanism for the development of GDM in lean Japanese women with BMI < 18.5 kg/m^2^, in whom islet cell antibodies were not detectable (8). The absence of antibodies in these women would be helpful to rule out type 1 DM.

### Diabetes in people with low BMI

**Figure 1:**
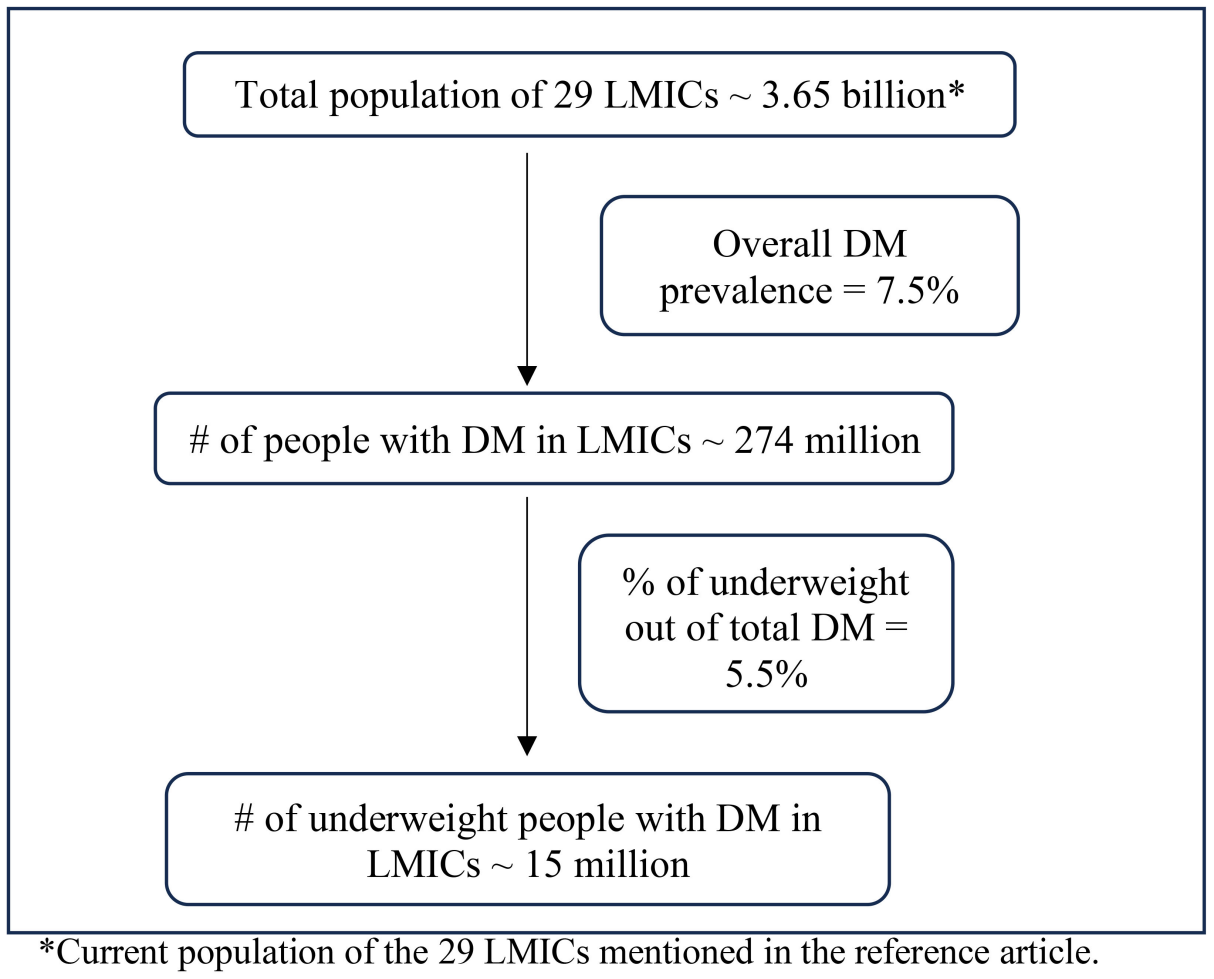
Prevalence of low BMI diabetes in low-middle-income countries. *Current population of the 29 LMICs mentioned in the reference article.

The growing body of literature on the metabolic characteristics of diabetes mellitus (DM) among people with low BMI could also help to shed light on the pathophysiology of GDM in lean women. To understand the metabolic characteristics of lean individuals with DM, Lontchi-Yimagou et al. conducted extensive metabolic studies in Vellore, India (11). Participants with low BMI and DM (in whom type 1 DM autoantibodies positive and monogenic forms of diabetes were carefully excluded) were compared to participants with type 1 DM, type 2 DM, and BMI-matched individuals without DM. Investigators used anthropometric measurements, hyperinsulinemic-euglycemic clamp studies (by maintaining the plasma glucose at ~90mg/dl and titrating optimal insulin infusion rates), and a standard Mixed-Meal Tolerance Test (MMTT) (to measure glucose, insulin, glucagon, and C-peptide levels after a standard meal followed by overnight fasting) to carefully phenotype these individuals. The researchers determined that the insulin secretory response in lean individuals with DM was markedly impaired; indeed, it was substantially lower than in subjects with type 2 DM, though higher than in subjects with type 1 DM. The indices for insulin resistance showed lower insulin resistance in lean DM participants compared to those with type 2 DM, but it did not differ significantly in type 1 DM patients (11). Consistent with these findings, Kibirige et al. used HOMA-IR measures to determine the metabolic characteristics of 568 Ugandan adults with newly diagnosed DM. The authors reported that leaner participants had significantly lower pancreatic β cell function than non-lean participants but did not demonstrate substantial insulin resistance (12). There have been dozens of articles (including India, Ethiopia, Japan, and Uganda) over many decades attesting to the persistent presence of diabetes among lean populations from different countries (13–23). Additionally, a recent meta-analysis based on nationally representative surveys that together totaled more than 0.5 million participants estimated the prevalence of low BMI diabetes to be 15 million in 29 low and middle-income countries (24) (Derived from the data as shown in **Figure 1**). Given this large projected number of people with low BMI diabetes, it is crucial to focus on this group of patients and dedicate further attention and research to this topic. This would help in understanding why lean women can also be susceptible to GDM.

### Postpartum risk of diabetes

#### Type 1 DM

Approximately 10% of women with GDM show autoantibody positivity during pregnancy or even for the first time after delivery (25). Clinically significant antibody levels (varying as per the ethnicity of the woman and type 1 DM risk in the background population) during pregnancy and postpartum can predict the development of type 1 DM postpartum (26, 27). The risk increases in women aged ≤ 30 years, those with more than two previous pregnancies, and those who required insulin during pregnancy (28, 29). Such women are at high risk of developing type 1 DM during pregnancy or postpartum or may develop a few years after delivery and evolve into Latent Autoimmune Diabetes of Adulthood (LADA) (25). So, women with GDM from high-risk populations should be screened for antibodies during pregnancy and followed up after delivery for early diagnosis of type 1 diabetes (30). It is reported that combined screening with three antibody markers [glutamic acid decarboxylase antibodies (GADA), islet cell antibodies (ICA), and antibodies to tyrosine phosphatase ICA512/IA-2 (IA2As)] increases the sensitivity to 82% compared to screening with only one or two antibodies (30).

#### Type 2 DM

Along with a progressive increase in the risk of type 2 DM in women with high BMI, intriguingly, lean women (with BMI<18 kg/m^2^) also show a relatively higher incidence of development of type 2 DM than women with normal BMI (31). Previous studies have reported that women with GDM have a ten times higher risk of developing type 2 DM than women without a history of GDM (32). The reason for the development of DM in these women is not yet fully understood; however, it is suggested to be due to pancreatic β-cell dysfunction in the presence of preexisting insulin resistance, unmasked during pregnancy (33).

It should be noted that most of the studies examining the association of GDM with the risk of type 1 or type 2 DM have focused on women with normal or high BMI rather than on lean women with GDM. So, it is crucial to conduct extensive longitudinal studies, potentially including such measures as fasting and postprandial glucose levels, C-peptide levels, and HOMA-IR to assess the risk in the latter population for early diagnosis and management.

### Complications and management

Given the unique clinical, epidemiological, and pathophysiological features of lean DM, the current treatment guidelines for overweight/obese women with GDM may not be helpful (or even potentially harmful) to lean women with GDM. For example, while large-for-gestational-age neonates are considered to be the most common adverse outcome in women with GDM, lean women with GDM are five times more likely to have small-for-gestational-age (SGA) neonates than normal or overweight women with GDM (34). Intrauterine protein deficiency has been clearly established to contribute to low birth weight and other neonatal development issues, as has been confirmed by many studies in animals (35). In addition, recommendations from Scandinavia have proposed that adequate protein intake is essential for the appropriate development of both fetal and maternal tissues during pregnancy (36). Management guidelines focused on carbohydrate and calorie limitation may further increase SGA and low birth weight. These conditions have long-term metabolic consequences for the neonate. Additionally, metformin, which is commonly used as a first-line pharmacologic therapy in GDM and increases maternal insulin sensitivity, may have limited efficacy in women whose primary deficit is insulin deficiency. Finally, insulin therapy may be dangerous in lean women because of food insecurity. Despite this, there is limited literature and recommendations focused on the specific management of GDM in lean women. Although the National Academy of Medicine (previously Institute of Medicine) (37), Voerman, et al. (38), and Luo et al. (39) do recommend greater gestational weight gain in underweight women than in women with normal/overweight/obese BMI (as shown in **Table 1**), additional research is needed to determine optimal protein and calorie intake for underweight women with GDM (40) as well as strategies to prevent postpartum diabetes in these individuals.

**Table 1:** Total gestational weight gain recommendations by NAM, Voerman et al., and Luo et al.

BMI category (kg/m^2^)	Total Gestational Weight Gain recommendation (in lb.) *		
	National Academy of Medicine	Voerman et al	Luo et al.
**Underweight (<18.5 kg/m^2^)**	28-40	30.9-<35.3	22-35
**Normal weight (18.5 - 24.9 kg/m^2^)**	25-35	22-<39.7*	17.7 - 26.2
**Overweight (25.0- 29.9 kg/m2)**	15-25	4.4-<35.3*	13.2 – 17.4
**Obese (≥30 kg/m2)**	11-20	4.4-<13.2^#^	-11 – 8.6^

*Rounded up to the nearest tenth.

#For obesity grade 1 (BMI 30-34.9 kg/m^2^). The article also includes optimal GWG for women.

with obesity in grade 2 (weight loss or weight gain of 0 to <8.8 lb.) and grade 3 (weight gain of 0 lb. to <13.2 lb.).

^The article recommends weight loss of 11 lb. to weight gain of 8.6 lb. during pregnancy for obese women.

## Conclusion

To conclude, although the prevalence of GDM among lean women is generally lower than in overweight and obese women, it is still a crucial area of study, given the high numbers of pregnancies among lean women globally. Indeed, lean pregnant women and their offspring face a unique set of challenges, including a higher risk of postpartum diabetes, as well as small-for-gestational-age neonates and preterm births. Thus, following the current guidelines might be detrimental to fetal outcomes, suggesting they may require specialized approaches to managing their GDM.

## Ethics statement

Ethical approval was not required for the study involving humans in accordance with the local legislation and institutional requirements. Written informed consent to participate in this study was not required from the participants or the participants’ legal guardians/next of kin in accordance with the national legislation and the institutional requirements.

## Author contributions

MH: Writing – review & editing. PW: Writing – original draft, Writing – review & editing.
